# Trivial Blunt Chest Trauma Leading to Acute Respiratory Distress Syndrome in a Child

**DOI:** 10.7759/cureus.42132

**Published:** 2023-07-19

**Authors:** Santosh K Rathia, Murugan TP, Varun Anand, Samreen Yusuf, Anil Kumar Goel, Pugazhenthan T

**Affiliations:** 1 Trauma and Emergency/Pediatric Emergency Medicine, All India Institute of Medical Sciences, Raipur, Raipur, IND; 2 Pediatric Emergency Medicine, All India Institute of Medical Sciences, Raipur, Raipur, IND; 3 Pediatrics, All India Institute of Medical Sciences, Raipur, Raipur, IND; 4 Pharmacology and Therapeutics, All India Institute of Medical Sciences, Raipur, Raipur, IND

**Keywords:** computed tomography, acute respiratory distress syndrome, acute lung injury, lung contusion, trivial, blunt thoracic trauma

## Abstract

Both blunt and penetrating chest trauma in children are less common than in adults but cause severe acute morbidity and mortality. As the literature suggests, pulmonary contusion is the most common chest injury in children, occurring in more than half of all blunt chest trauma cases. Even patients with blunt injuries are likely to have a longer hospital stay. The difference in physiological and anatomical variations in children compared to adults makes it more difficult from the diagnosis, management, and monitoring perspectives. A thorough physical examination is needed with close clinical monitoring, and additional vigilance is important during the management of a child. The physiologic consequences, such as the dreaded complication of alveolar hemorrhage and pulmonary parenchymal destruction, usually manifest within a few hours of the trauma and can take up to seven days to recover. Hence, timely diagnosis is crucial during the emergency evaluation. The clinical diagnosis can be supported by a special imaging modality in the form of chest computed tomography (CT), which confirms the radiological parenchymal destruction with high sensitivity. Management is mostly supportive to start with and includes high-flow oxygen, ventilatory pressure support as needed for the severity of acute lung injury (ALI) or acute respiratory distress syndrome (ARDS), judicious fluid administration, control of the pain associated with bony and thoracic soft tissue injuries, and careful hemodynamic monitoring for other complications and sequelae likely to develop.

Here, we report an interesting case of a 10-year-old male child presenting to the Pediatric Emergency Department with acute moderate-to-severe respiratory distress that developed after two days of a few vomiting episodes along with non-specific lower chest and substernal pain following blunt trauma to the chest. The injury was trivial in nature as described by the father caused by an accidental fall on a small pile of bricks while playing near his home. After triaging under the red category, the child was managed in line with acute respiratory distress. We ruled out pneumothorax, hemorrhagic pleural effusion or pericardial effusion, and other evidence of invasive chest as well as gross abdominal injuries by comprehensive but focused history and clinical examinations, including adjuncts such as point-of-care ultrasound) and chest X-ray (CXR). Although the initial arterial blood gas analyses were suggestive of a mild form of ARDS or ALI by the criteria based on the P:F ratio (PaO_2_ to FiO_2_ ratio, which was between 200 and 300 for the case), the CXR and the chest CT revealed that the child had significant lung parenchymal injury in the form of bilateral fluffy pulmonary infiltrates. This case indicates that even a trivial blunt trauma can induce certain mechanisms of lung injury, leading to severe manifestations and sometimes fatal complications such as pulmonary contusion, hemorrhage, and ARDS.

## Introduction

Pulmonary contusions are lung injuries with alveolar destruction usually sustained during blunt injury to the chest. The physiologic consequences of alveolar hemorrhage and pulmonary parenchymal destruction usually last for a week. They may clinically present with respiratory distress, hypoxemia, and hypercarbia [1]. Children are more likely to develop hypoxemia compared to adults considering their lesser functional residual capacity [2]. Prompt and timely decision-making is needed between clinical suspicion and diagnostic confirmation. Although most of the management approaches remain supportive, they target improving oxygenation and effective ventilation to maintain other acute vital physiologies, as well as to prevent immediate complications and the future risk of prolonged structural or functional sequelae. Acute lung injury (ALI) or acute respiratory distress syndrome (ARDS) might be the most dreaded complication of an acute traumatic chest injury. Most trauma reports on children include both blunt injury and penetrating injury to the chest as etiologies for noticeable lung damage, including pulmonary hemorrhage, contusion, or ARDS, but, usually, they are associated with moderate-to-severe trauma [3].

Here, we report a minor or trivial blunt chest trauma leading to significant lung parenchymal injury culminating in severe clinical manifestations as well as an overtly evident radiological picture of bilateral pulmonary contusions.

## Case presentation

A 10-year-old male child presented to our Pediatric Emergency Department (ED) with symptoms of mild chest pain localized to the substernal region and five to six episodes of vomiting for the last two days with gradual worsening of breathlessness over 12 hours. He was injured due to an accidental fall on a pile of bricks three days ago while playing near his home. The fall was trivial and there were no obvious abrasions or other skin injury marks on his chest, which was the least of the concerns for the parents for the initial two days.

On presentation to the pediatric ED, the child was quickly triaged into the red category by the Pediatric Assessment Triangle evaluation considering increased breathing and an irritable and anxious look. After immediately shifting to the red zone resuscitation area, a further primary examination was done following the standard ABCDE (airway, breathing, circulation, disability, and exposure) approach. The airway was open and maintainable, but the child was in respiratory distress with severe hypoxia, having a room air oxygen saturation of 74% with tachycardia (heart rate of 152 beats/minute), tachypnea (respiratory rate of 48 breaths/minute), and subcostal and intercostal retractions. On auscultation, he had slightly decreased air entry bilaterally, with diffuse crepitations noted mainly in the bilateral inframammary and axillary areas. Although the child was afebrile during this evaluation, he had non-specific lower chest pain with a pain score of 4-6 on the Wong-Baker Face scale. There were no signs of circulatory compromise, and a focused examination revealed no evidence of involvement of other systems or a breach in physical integrity from head to toe.

**Figure 1:**
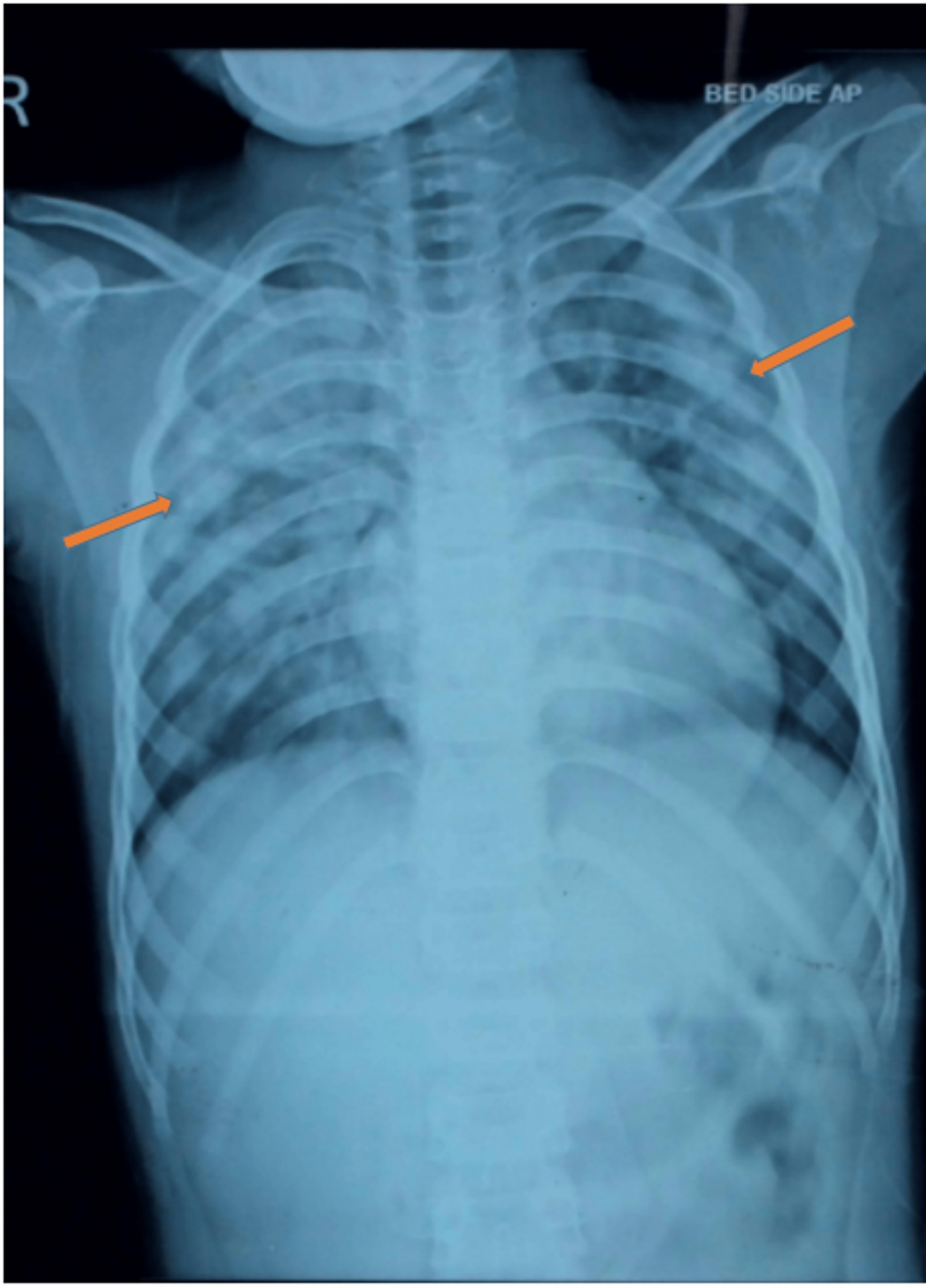
Chest X-ray revealing bilateral fluffy opacities (shown by orange markers) suggestive of pulmonary contusions. Radiological opacities suggested lung contusions according to the radiologist’s reporting for the clinical setting of antecedent blunt thoracic trauma.

**Figure 2:**
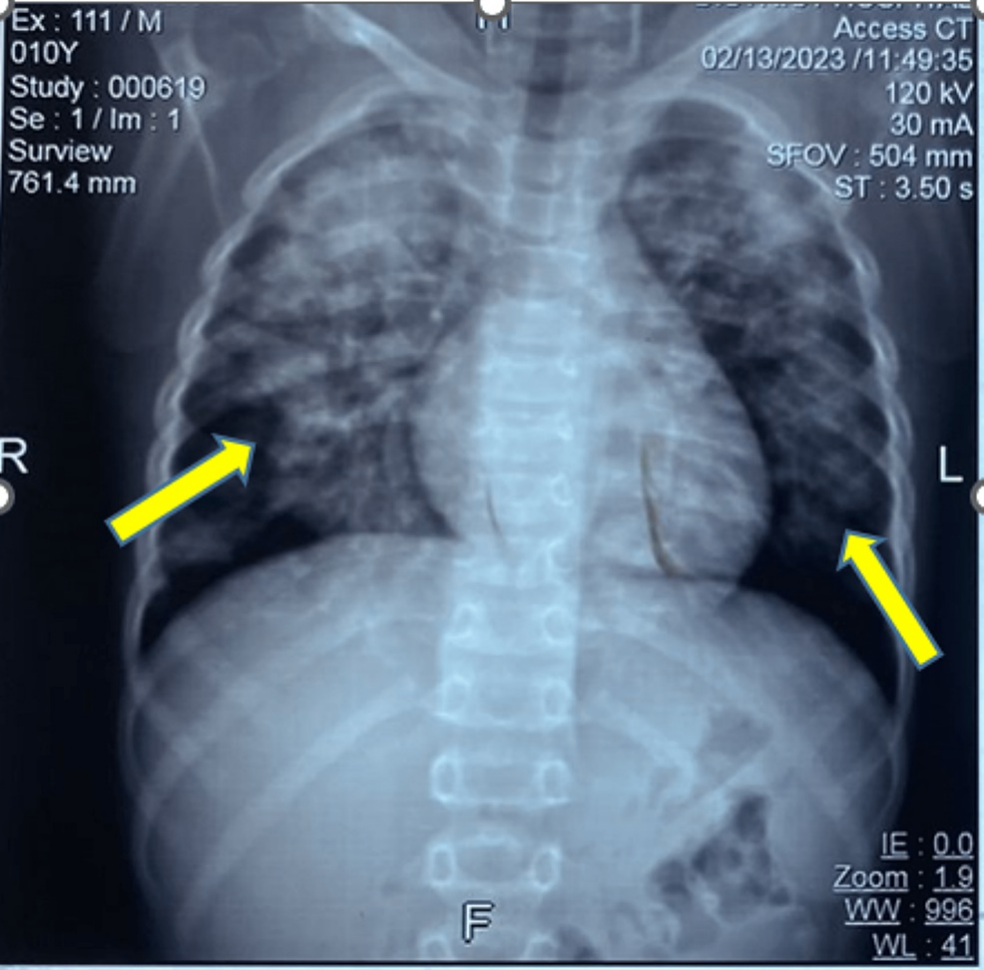
Computed tomography of the thorax (scout film) showing bilateral multifocal patchy hyperdensities.

**Figure 3:**
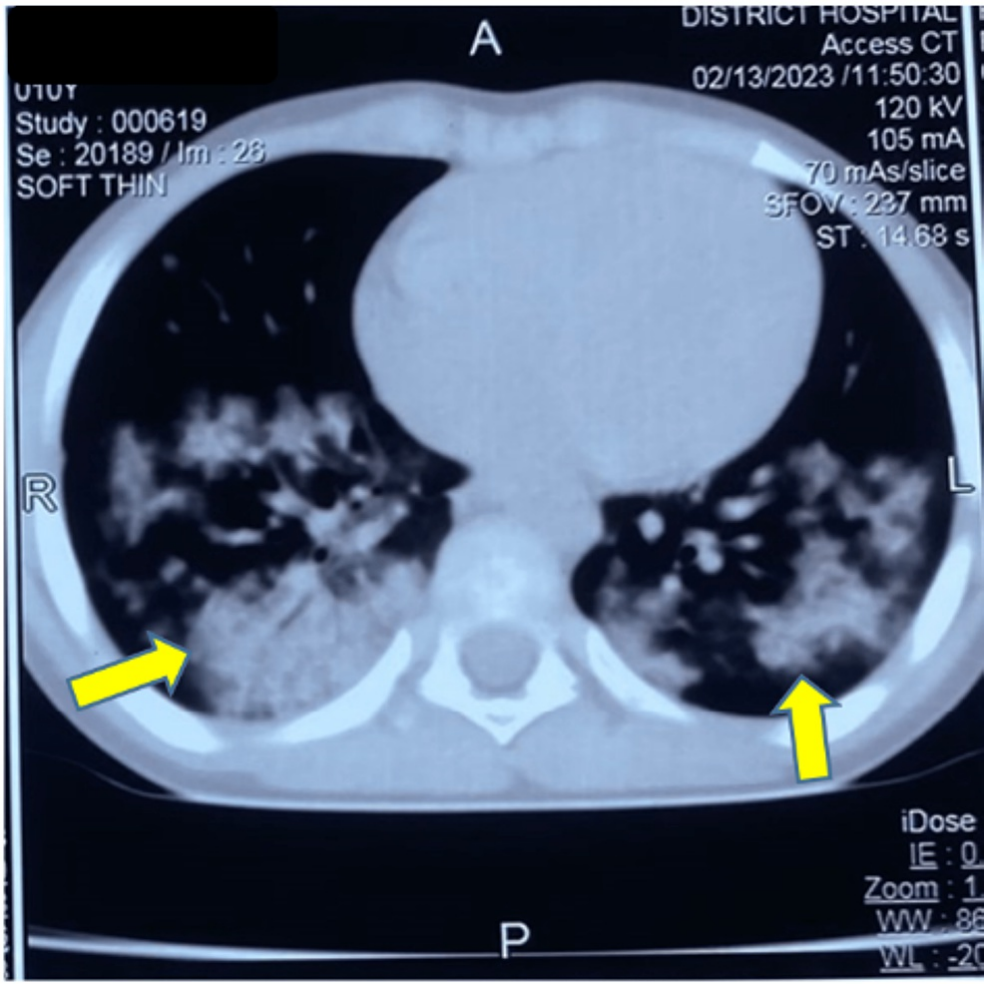
Computed tomography of the chest showing bilateral patchy hyperdense lesions in lung parenchyma.

**Figure 4:**
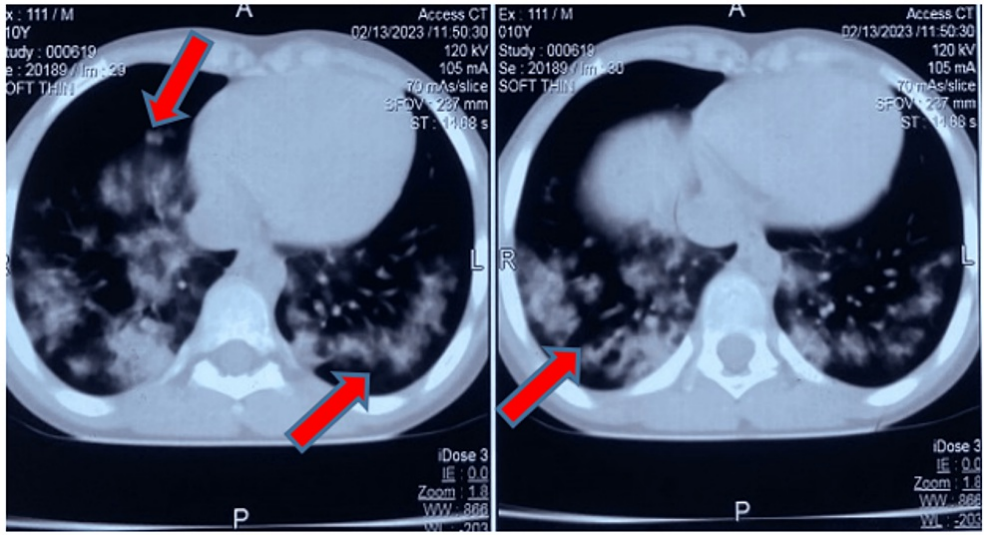
Contrast-enhanced computed tomography of the chest. Red arrows show multifocal confluent hyperdense lesions present in bilateral lower lung segments.

To rule out acutely life-threatening injuries such as pneumothorax, hemorrhagic pericardial effusion or tamponade, and other invasive thoracic as well as abdominal injuries beyond a comprehensive but focused clinical examination, important diagnostic adjuncts such as point-of-care ultrasound modality for extended-FAST (an extended form of focused abdominal sonography in trauma patients), arterial blood gas (ABG), chest X-ray (CXR), and computed tomography (CT) were performed. Although the initial ABG analyses were suggestive of a mild form of ALI or ARDS by the criteria based on the P:F ratio (PaO_2_ to FiO_2_ ratio which was between 200 and 300 for the index case), the CXR and the chest CT revealed that the child had significant lung parenchymal injury in the form of bilateral fluffy pulmonary infiltrates (Figures 1-4).

After an initial assessment by primary and secondary surveys, the clinical impression of blunt thoracic trauma to the lower anterior chest wall was considered. Subsequently, by clinical elimination, other possibilities such as pneumonia, sepsis, shock, aspiration, fluid overload, or any blood product-related or transfusion-associated ALI were ruled out. As the preliminary chest radiograph showed non-specific bilateral patchy opacities, the chest CT scans (Figures 2-4) and their formal reports by the radiologist consultant of the institute confirmed the diagnosis of pulmonary contusion.

The patient was initially stabilized with a high-flow oxygen device, and other symptomatic treatments such as analgesics, antiemetics, proton-pump inhibitors, and antibiotics were also started in the ED. Initial blood gas revealed a P:F ratio of 207, suggestive of ALI. Subsequently, after the high-flow oxygen therapy, the ratio improved to 270 during the ED stay with normal SpO_2_ and was ultimately normalized on elective non-invasive ventilation (NIV) support, which had been initiated before a shift to the pediatric intensive care unit (PICU) on day two to combat persistent mild tachypnea and retractions so that the lungs would be allowed to heal. Other point-of-care investigations, including sepsis workup and inflammatory markers, were negative and did not reveal any evidence of infection. However, he was empirically started on a broad-spectrum antibiotic. Over the course of his stay in the ED and after shifting to the PICU on NIV support, his clinical condition gradually improved, and his distress settled with a normal P:F ratio and other blood gas parameters. Finally, oxygen support was tapered and weaned off, and the patient was discharged on the sixth day from the pediatric ward in a clinically stable condition.

## Discussion

As discussed in the index case, the child presented with hypoxemia and an initial decreased P:F ratio with evidence of ALI or a mild or evolving form of ARDS, which subsequently required high-flow oxygen delivery. As the child had sustained a trivial fall with unnoticeable chest trauma and had developed bilateral lung contusions with evident radiological and clinical manifestations of significant ALI, the presentation was remarkable and quite atypical. Hemorrhage into the pulmonary parenchyma leads to contusion pathophysiology that worsens for 24-48 hours and then generally resolves seven days after the injury [1]. The impact of injury should be severe enough to cause disruption of the capillaries of the alveolar walls and septa, causing leakage of blood into the alveolar spaces and interstitium [4], which in our case was very unusual and difficult to explain with such an impact of injury. The pain associated with the injury sustained could be involved in splinting, which may further restrict ventilation and cause atelectasis, which could have been a possibility in our index case. As revealed by Western studies, respiratory distress usually worsens at or after 48-72 hours, depending on injury severity, and vanishes gradually in a few days [1-4]. A similar natural course was observed in our child, who presented on day three of trauma with breathlessness. Patients usually have other associated features such as hemoptysis and chest pain.

However, the diagnosis of pulmonary contusion remains very challenging. CXR could miss the subtle changes, especially if done early in the disease course (in a study, the sensitivity of X-ray was 47% at admission versus 92% after 24 hours of trauma), while CT of the thorax remains the standard diagnostic modality of choice, with a sensitivity of up to 100% and a specificity of around 40%. Chest CT remains superior for accurate assessment in the diagnosis of pulmonary contusions secondary to blunt chest injury [1]. A typical chest CT finding highlighted in a study was subpleural sparing, which can be observed in 95% of children with pulmonary contusions but is not seen in children with atelectasis or pneumonia [5]. The same study also mentioned that lung contusions tend to be posterior (60%), crescentic (50%), amorphous (45%), and have confluent or nodular components (70%). However, in 2016, Golden et al. stated that the use of chest CT should be limited to the identification of intrathoracic vascular injuries when confronted with an abnormal mediastinal silhouette on CXR [6].

The treatment of pulmonary contusions remains majorly supportive with good oxygenation and adequate analgesia support. There is usually a very minimal need for invasive and mechanical ventilation [7]. Rather, one can raise the question of whether or not an NIV modality would help in early recovery and treating obstructive atelectasis. As in our case, high-flow oxygen and non-invasive positive-pressure support helped the child recover faster.

Pulmonary contusion is the most common chest injury in children, occurring in more than half of all blunt chest traumas [8], and many victims do not have evidence of external chest wall trauma [9]. We noted similar findings in our unique observation in the index case, which presented with no noticeable external injuries.

The risk factors for developing trauma-associated ARDS may include direct lung injury, direct chest wall injury, hemorrhagic shock, massive transfusion, aspiration, pneumonia, secondary sepsis, severe traumatic brain injury, and quadriplegia [10,11]. Although often no clear-cut mechanism or risk factor might be identified, which causes ALI or ARDS following a trivial form of blunt chest trauma, we must adequately monitor and follow patients for possible causal risk factors and likely complications of acute lung insults caused by thoracic trauma.

## Conclusions

With blunt trauma to the chest, lung contusions are the most common injury in children. This is due to the compliant chest wall of young patients, which allows significant parenchymal injury even without bony injury. Most cases of blunt chest trauma would resolve spontaneously, but some may develop ARDS. Chest CT is considered the imaging modality of choice compared to CXR in view of its higher sensitivity and acceptable specificity in diagnosing lung contusions. Treatment is mostly symptomatic and supportive to sustain effective oxygenation and ventilation.
